# Human Umbilical Cord Mesenchymal Stem Cell–Derived Exosomes Attenuate Renal Fibrosis by Suppressing Fibroblast Activation via the INHBA/PI3K/AKT Pathway

**DOI:** 10.1002/pdi3.70061

**Published:** 2026-06-29

**Authors:** Meiling Chen, Chenxi Jia, Zhuocheng Shi, Bianan Zhu, Yihang Yu, Xing Liu, Feng Liu, Jianwei Chen, Tao Xu, Yuanyuan Zhang, Deying Zhang, Guanghui Wei

**Affiliations:** ^1^ Ministry of Education Key Laboratory of Child Development and Disorders, Department of Urology Children's Hospital of Chongqing Medical University, National Clinical Research Center for Child Health and Disorders Chongqing China; ^2^ Chongqing Key Laboratory of Structural Birth Defect and Reconstruction Chongqing China; ^3^ Children Urogenital Development and Tissue Engineering of Chongqing Education Commission of China Chongqing China; ^4^ Department of Pediatric Urology West China Second University Hospital, Sichuan University Chengdu Sichuan China; ^5^ Key Laboratory of Birth Defects and Related Diseases of Women and Children (Sichuan University), Ministry of Education Chengdu Sichuan China; ^6^ Center for Bio‐Intelligent Manufacturing and Living Matter Bioprinting Research Institute of Tsinghua University in Shenzhen, Tsinghua University Shenzhen Guangdong China; ^7^ Wake Forest Institute for Regenerative Medicine, Wake Forest School of Medicine Winston‐Salem North Carolina USA

**Keywords:** exosomes, fibroblast activation, INHBA, mesenchymal stem cells, PI3K/AKT signaling pathway, renal fibrosis

## Abstract

Renal fibrosis is a common pathological feature and key driver of progression to end‐stage renal disease in various chronic kidney diseases, with effective treatments remaining scarce. Exosomes derived from mesenchymal stem cells (MSC‐Exos) have demonstrated tremendous potential in tissue and organ repair and antifibrosis treatment. This study investigated the therapeutic effects and mechanisms of MSC‐Exo derived from human umbilical cord (HucMSC‐Exo) on renal fibrosis. HucMSC‐Exo was applied to intervene in a mouse model of renal fibrosis induced by unilateral ureteral obstruction (UUO) or cocultured with TGF‐β‐stimulated rat renal fibroblasts (NRK‐49F). Results showed that HucMSC‐Exo conspicuously alleviated pathological damage, inflammatory response, fibroblast proliferation/activation, and extracellular matrix deposition in UUO kidneys. Single‐cell sequencing revealed a prominent upregulation of *Inhba* gene in UUO kidneys, whereas HucMSC‐Exo treatment effectively inhibited its expression. Further mechanistic studies showed that knocking down *Inhba* mimicked the antifibrotic effects of HucMSC‐Exo, whereas exogenously adding INHBA weakened its protective effects. Transcriptome sequencing results revealed that the downstream effects of *Inhba* involve regulating the PI3K/AKT signaling pathway, and HucMSC‐Exo treatment markedly inhibited the activation of this pathway. Collectively, HucMSC‑Exo exerts antifibrotic effects by regulating the INHBA/PI3K/AKT signaling axis to inhibit renal fibroblast activation.

## Graphical abstract

MSC‐Exo derived from human umbilical cord (HucMSC‐Exo) downregulates INHBA expression, consequently modulating the PI3K/AKT signaling cascade. This regulation attenuates renal fibroblast activation and extracellular matrix deposition, thereby exerting a renoprotective effect against fibrosis. UUO, unilateral ureteral obstruction; MSC‐Exo, Exosomes derived from mesenchymal stem cells.

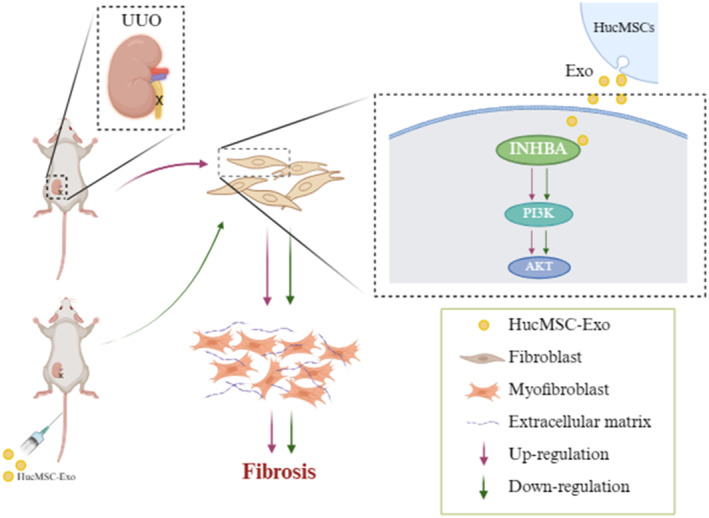

## 1 Introduction

Due to unique physiological microenvironment, the kidney is highly susceptible to factors such as ischemia, hypoxia, or infection. With improvements in global living standards and aging populations, the incidence of kidney diseases has been conspicuously increasing [1, 2]. Acute kidney injury (AKI) and chronic kidney disease (CKD) are the two primary categories of kidney diseases, posing a serious threat to human health. Acute kidney injury (AKI) is a common and serious complication in hospitalized patients, with a reported in‐hospital incidence of 12.2% and an associated crude mortality rate of approximately 10%, as demonstrated in a large multicenter study. The global burden of AKI is substantial and continues to rise. Meanwhile, the global prevalence of CKD exceeds 10%, with a total of 840 million cases, and it is projected to become the fifth leading cause of death globally by 2040 [3, 4, 5]. Historically, AKI and CKD were regarded as distinct clinical conditions; however, accumulating evidence now demonstrates that they are pathophysiologically interconnected. CKD increases susceptibility to AKI, whereas AKI accelerates the onset and progression of CKD. Both conditions may ultimately lead to end‐stage renal disease (ESRD), dramatically impairing long‐term renal outcomes and the patients' quality of life [6, 7].

The transition from AKI to CKD involves complex and interconnected pathological processes, including tubular epithelial cell injury, interstitial fibrosis, persistent inflammation, and endothelial dysfunction. Following renal injury, surviving tubular epithelial cells may proliferate and dedifferentiate to restore tubular integrity and function, whereas resident fibroblasts and pericytes are activated to synthesize extracellular matrix (ECM) components that contribute to tissue repair. However, repeated or sustained injury results in maladaptive repair, characterized by incomplete tubular regeneration and progressive ECM accumulation [8, 9, 10, 11]. Renal interstitial fibrosis—defined as abnormal deposition of ECM components, such as fibronectin and collagen, within the renal interstitium—is a hallmark of pathological feature and final common outcome of CKD [12]. Myofibroblasts are the principal effector cells responsible for ECM production, approximately 50% of myofibroblasts originate from resident fibroblasts, whereas the remainder derives from pericytes, endothelial cells, tubular epithelial cells, and macrophages [13, 14, 15]. Despite advances in our understanding of renal fibrosis mechanisms, effective antifibrotic pharmacotherapies have yet to be realized, underscoring the urgent need for innovative therapeutic approaches.

Mesenchymal stem cells (MSCs) are among the most extensively studied stem cell types in regenerative medicine, with multiple tissue sources including adipose tissue, bone marrow, umbilical cord, and placenta. They exert protective effects across a wide range of diseases, including joint disorders, neurological diseases, dental tissue damage, and cancer [16, 17, 18, 19]. Although earlier studies attributed the therapeutic benefits of MSCs to direct engraftment and differentiation at sites of injury, subsequent investigations revealed that MSCs act primarily through paracrine mechanisms. Extracellular vesicles, particularly exosomes, are now recognized as the principal mediators of MSC therapeutic effects. Exosomes are nanosized vesicles (30–150 nm) that carry proteins, nucleic acids, and lipids reflective of their parent cells, encapsulating much of the biological potency of MSCs. Compared with MSC transplantation, exosome‐based therapy offers several advantages, including greater safety, reduced immunogenicity, minimal risk of immune rejection, and enhanced stability in storage and transport. Consequently, exosomes are increasingly viewed as a promising cell‐free alternative to conventional stem cell therapy [20, 21, 22].

Activin A, a member of the transforming growth factor‐β (TGF‐β) superfamily, is composed of two inhibin βA subunits encoded by the *Inhba* gene, linked by disulfide bonds. Activin A plays a pivotal role in immune regulation, inflammation, cell proliferation, differentiation, fibrosis, and tumorigenesis [23].

Here, we successfully established a renal fibrosis model through unilateral ureteral obstruction (UUO) and treated fibrotic kidneys with exosomes derived from HucMSC‐Exo. The results of single‐cell RNA sequencing revealed that the *Inhba* gene was effectively inhibited by HucMSC‐Exo in the UUO model. Through transcriptomic sequencing, we explored the specific mechanisms by which INHBA promotes fibrosis development. Furthermore, we elucidated that HucMSC‐Exo suppress fibroblast differentiation via regulating the PI3K/AKT pathway through INHBA, thereby achieving a fibrotic‐relieving effect.

## 2 Materials and Methods

### 2.1 Animal Experiments

Animal experiments in the study were approved by the Animal Research Committee of Chongqing Medical University (no. CHCMU‐IACUC20250429004). Thirty adult male C57BL/6 mice were purchased from Chongqing Medical University (license no. SYXK [Chongqing] 2007‐0001) and housed in the Experimental Animal Center of Children's Hospital of Chongqing Medical University (license no. SYXK [Chongqing] 2007‐0016). The mice were raised in polycarbonate cages with free access to food and water, under a 12‐h light/dark cycle and a constant room temperature of 24°C, with a relative humidity of 60%.

Animals were randomly divided into five groups (*n* = 6 in each group): Sham, UUO3, UUO7, UEK3 (UUO3 + Exo, U: UUO; E: Exo; K: kidney), and UEK7 (UUO7 + Exo). In the UUO groups, under sterile conditions and isoflurane anesthesia, the upper one‐third of the left ureter was permanently ligated with a 4–0 silk suture. Left kidneys were collected on postoperative day 3 (UUO3 group) or day 7 (UUO7 group) under 2% isoflurane anesthesia. Mice in the sham group underwent the same procedure except for ureteral ligation. In the UEK3 group, HucMSC‐Exo (100 μg) [24, 25] was injected via the tail vein on UUO postoperative day 1 and left kidneys were collected on postoperative day 3. In the UEK7 group, HucMSC‐Exo (100 μg per injection) was injected via the tail vein on UUO postoperative days 1 and 3 and left kidneys were collected on postoperative day 7. Harvested kidneys were partially fixed in 4% paraformaldehyde, and the remaining tissue was stored at −80°C for subsequent experiments.

### 2.2 Tissue Dissociation and Single‐Cell RNA Sequencing

The harvested kidney tissue was transferred to a culture dish containing 1 × phosphate‐buffered saline (PBS) (RNase‐free, without Ca^2+^/Mg^2+^), cut into 0.5 mm^2^ pieces, and washed repeatedly with 1 × PBS. The tissue pieces were incubated with a dissociation solution (0.35% collagenase IV, 2 mg/mL papain, and 120 U/mL DNase I) for 20 min. Dissociation was terminated by adding 1 × PBS supplemented with 10% fetal bovine serum (FBS). The resulting cell suspension was filtered through 70 and 30 μm cell strainers, then centrifuged at 300 × g for 5 min at 4°C. The cell pellet was collected and resuspended in 100 μL of 1 × PBS containing 0.04% bovine serum albumin (BSA). Red blood cells were removed by adding 1 mL of red blood cell lysis buffer for 2–10 min. After dead cell removal, the cells were finally resuspended in 50 μL of 1 × PBS (0.04% BSA).

The resulting single‐cell suspension was loaded onto the 10 × Chromium platform. Cell capture, cDNA amplification, and library construction were performed according to the manufacturer's instructions for the 10 × Genomics Chromium Single‐Cell 3′ kit (V3). The constructed libraries were sequenced on the NovaSeq 6000 platform in a paired‐end multiplexing run (150 bp).

### 2.3 Single‐Cell RNA‐Seq Data Processing and Analysis

Raw sequencing data were demultiplexed and converted to FASTQ format using Illumina bcl2fastq (version 2.20). Quality control and alignment for each sample were performed using cell ranger (version 3.1.0, 10 × Genomics). Downstream analysis, including dimensionality reduction, clustering, and expression matrix analysis, was carried out with Seurat (version 4.1.0). Visualization was performed using uniform manifold approximation and projection (UMAP) and t‐distributed stochastic neighbor embedding (tSNE) implemented in Seurat. Cell types for each cluster were annotated based on canonical marker genes from published literature and online databases. Differentially expressed genes were identified using the “FindMarker” function in Seurat with a threshold of log_2_ FC ≥ 0.25 and an adjusted *p* value < 0.01.

### 2.4 Cell Culture

NRK‐49F fibroblasts (Normal Rat Kidney‐49F; China Center for Type Culture Collection) were maintained in Dulbecco's Modified Eagle Medium Nutrient Mixture F‐12 (DMEM/F12, Sigma‐Aldrich, MO, USA) containing 10% FBS (ZETA LIFE, CA, USA) and 100 U/mL penicillin and 100 mg/mL streptomycin, with culture conditions of 37°C, 5% CO_2_, and 100% humidity. After NRK‐49F cells had grown to approximately 80%–90% confluence, recombinant human transforming growth factor β1 (TGF‐β1) was added to the culture at a final concentration of 10 ng/mL in the absence or presence of HucMSC‐Exo (100 μg/mL) with 48 h. The morphology of NRK‐49F cells was examined using a light microscope (Olympus Corporation, Tokyo, Japan).

HucMSCs (cat. no. PCS‐500‐010) were provided by Chongqing Stem Cell Biotechnology R&D Base, Chongqing, China. Additionally, the cultivation of the cell, collection of the supernatant, and the extraction/identification of HucMSC‐Exo were carried out as described previously [24].

### 2.5 RNA Sequencing Analysis

Four replicate NRK‐49F cell samples were collected from each group for high‐throughput mRNA sequencing on Illumina NovaSeq 6000 platform. *p* value < 0.05 and |logFC| > 2 were used as the screening conditions and the samples in each group were analyzed for differences using the R software. Cluster analysis, volcano mapping, Gene Ontology (GO), and Kyoto Encyclopedia of Genes and Genomes (KEGG) enrichment analyses of differential genes were conducted on the Tsingke Cloud platform (https://www.tsingke.com.cn). Meanwhile, gene set enrichment analysis (GSEA) was performed to explore potential pathway and a *p* value < 0.05 was considered statistically significant.

### 2.6 Cell Transfection

Three siRNAs (siRNA1/2/3) for *Inhba* were purchased from the Tsingke Biotechnology (Supporting Information S1: Table S1). Cells were seeded into culture plates until the cell confluence reaches 30%–40%. Then, the diluted *Inhba* siRNA and negative control siRNA with the transfection reagent Lipofectamine 3000 were mixed in a specific ratio and let it stay at room temperature for 20 minutes to form a complex. The mixture was added to serum‐free cell culture medium (opti‐MEM, Thermo Fisher, MA, USA) and incubated at 37°C for 6–8 hours. Then, it was replaced with fresh culture medium and added TGF‐β1 for intervention. Samples were collected after 48 hours to detect the expression of relevant indicators.

### 2.7 Histological Observations

Kidney tissue samples were fixed in 4% paraformaldehyde for 48 h. Subsequently, the samples were dehydrated through a graded ethanol series: 75% ethanol for 100 min, 85% ethanol for 80 min, and 95% ethanol for 80 min (repeated three times), followed by 100% ethanol for 60 min (repeated two times). The tissues were then cleared in xylene for 30 min (repeated three times), and infiltrated with paraffin wax for 30 min (repeated three times). Finally, the samples were embedded in paraffin and sectioned at a thickness of 4 μm. The sectioned specimens were subjected to dewaxing and gradient alcohol treatment, hematoxylin–eosin (H&E), and periodic acid–Schiff (PAS) staining were performed to evaluate the extent of damage to the renal tissue. Also, the Masson trichrome staining kit (Leagene, Beijing, China) was applied to assess collagen deposition according to the manufacturer's instructions.

### 2.8 Immunohistochemical Staining

The above paraffin sections were deparaffinized and treated with gradient alcohol, then placed in citrate buffer for microwave repair, followed by preparation of a 3% concentration of hydrogen peroxide solution to seal the endogenous peroxidase for 10 minutes. Next, the samples were blocked with 5% BSA for 1 hour and coincubated with the corresponding primary antibody at 4°C overnight. The next day, these sections were incubated with the corresponding 1:200 secondary antibody for 1hour and positively stained using 3,3′‐diamnobenzidine (DAB, Abcam, MA, USA), which was seen to give a tan expression in the positive areas. Finally, hematoxylin was used for counterstaining, and then the slices were sealed. The visualization method was consistent with H&E staining.

### 2.9 Immunofluorescence Staining

Cells were seeded in 24‐well plates containing crawler sheets and treated as described previously. After the intervention, the cells were washed with PBS three times, followed by fixation with 4% paraformaldehyde for 15 minutes and sealing with 5% BSA for 1 hour. Subsequently, these cells were incubated overnight at 4°C with different primary antibodies. The next day, they were individually treated with the corresponding fluorescent secondary antibodies and Hoechst, then washed three times with PBS buffer and sealed with an antifluorescence quencher.

Kidney tissue sections were obtained according to the above method, and the same process was implemented as for immunohistochemistry before incubation with secondary antibodies. Next, the tissue slides were coincubated with fluorescent secondary antibodies of the corresponding species at room temperature for 1 hour and washed three times with PBS buffer on a shaker. After that, incubated with Hoechst for 1 hour, the cells were washed three times with PBS buffer, and finally blocked with an anti‐fluorescent quencher. Finally, the images were acquired by confocal microscope (Nikon, Tokyo, Japan), analyzed and processed by the NIS Viewer software.

### 2.10 Cell Counting Kit‐8 (CCK‐8) Assay

Cell proliferation was evaluated by the CCK‐8 assay (CCK‐8; Dojindo, Kumamoto, Japan). A total of 3000 NRK‐49F cells were seeded in each 96‐well plate and treated with INHBA at concentrations of 0, 2.5, 5, 10, 20, and 40 ng/mL for 48 hours, respectively. Subsequently, 100 μL of the serum‐free culture medium containing 10% CCK‐8 stock solution was added to each well and incubated at 37°C for 2 hours, followed by measurement of the absorbance at 450 nm by enzyme‐linked immunosorbent assay plate reader (Olympus Corporation, Tokyo, Japan).

### 2.11 Real‐Time Polymerase Chain Reaction (PCR)

Total RNA was extracted from frozen kidneys and NRK‐49F cells using TRIzol Reagent. Following the manufacturer's protocol, 1 μg of RNA was reversely transcribed into cDNA using RT Master Mix for quantitative PCR (qPCR; MCEs, NJ, USA). Gene expression levels were then quantified by real‐time PCR using SYBR Green Master Mix (Tiangen, Beijing, China) on a CFX96 system (Bio‐Rad, CA, USA), with GAPDH serving as the endogenous control. Relative mRNA expression was calculated using the 2^−ΔΔCt^ method. Primer sequences are detailed in Supporting Information S1: Table S2.

### 2.12 Western Blot

Protein extracts were prepared from kidney tissues and NRK‐49F cells using RadioImmunoPrecipitation Assay (RIPA) Lysis Buffer (Beyotime, Shanghai, China) supplemented with 1% Phenylmethylsulfonyl Fluoride (PMSF, Solarbio, Beijing, China). Following protein quantification via BCA assay, samples were dissolved in 5 s× loading buffer, resolved by Sodium Dodecyl Sulfate PolyAcrylamide Gel Electrophoresis (SDS‐PAGE), and electroblotted onto Polyvinylidene Difluoride (PVDF) membranes (Millipore, MA, USA). Membranes were blocked at room temperature for 10 minutes using a rapid blocking solution (NCM, Jiangsu, China) prior to overnight incubation at 4°C with the designated primary antibodies (details in Supporting Information S1: Table S3). Subsequently, membranes were probed with corresponding secondary antibodies (Zenbio, Chengdu, China) for 1 hour at room temperature. Protein bands were visualized using enhanced chemiluminescence (ECL; Bio‐Rad, CA, USA) and quantified with ImageJ software.

### 2.13 Statistical Analysis

All experimental data were analyzed using GraphPad Prism 8.0 and expressed as mean ± standard deviation (SD). Data between two groups were compared by unpaired *t*‐tests and multiple groups were compared by one‐way analysis of variance (ANOVA). All experiments were performed independently three times and a *p* value < 0.05 was considered statistically significant.

## 3 Results

### 3.1 HucMSC‐Exo Ameliorates Renal Pathology and Inflammation in UUO Mice

**FIGURE 1:**
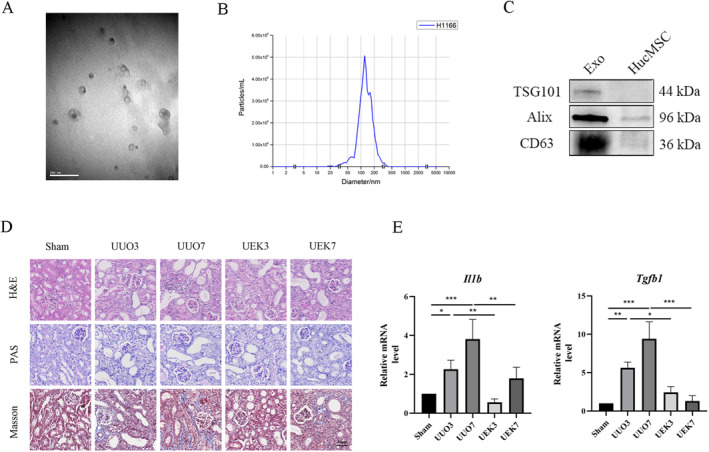
MSC‐Exo derived from human umbilical cord (HucMSC‐Exo) alleviates unilateral ureteral obstruction (UUO)‐induced kidney injury, inflammation, and fibrosis. (A) Observation of the morphology and structure of HucMSC‐Exo by electron microscopy; (B) detection of the diameter distribution of HucMSC‐Exo by particle size analysis; (C) detection of the expression of HucMSC‐Exo and HucMSC‐related markers by Western blot; (D) representative images of kidney hematoxylin‐eosin (H&E), periodic acid‐Schiff (PAS), and Masson staining; (E) mRNA expression levels of IL‐1β and TGF‐β1 in the kidney analyzed by real‐time quantitative polymerase chain reaction. *p < 0.05; **p < 0.01; ***p < 0.001. IL‐1β, interleukin‐1β; MSC‐Exo, Exosomes derived from mesenchymal stem cells; TGF‐β1, transforming growth factor‐β1; UEK3, UUO3+Exo; UEK7, UUO7+Exo.

Characterization of the extracted HucMSC‐Exo by electron microscopy, particle size analysis, and western blotting revealed that they exhibited a bilayer membrane structure, a peak particle size between 100 and 150 nm, and high expression of the exosome markers TSG101, Alix, and CD63 (Figure 1A–C). To assess renal morphological alterations across different mouse models, kidney sections underwent H&E and PAS staining. Comparative analysis revealed that, relative to Sham controls, UUO‐induced kidneys exhibited progressive pathological changes over time. These included tubular dilation/atrophy, tubular epithelial cell necrosis and sloughing, interstitial inflammatory cell infiltration, and tubular basement membrane thickening. Importantly, intervention with HucMSC‐Exos markedly attenuated both the severity and extent of these renal injuries and associated inflammation (Figure 1D,E).

### 3.2 HucMSC‐Exo Inhibits Kidney Fibroblast Activation and Fibrosis in UUO Mice

**FIGURE 2:**
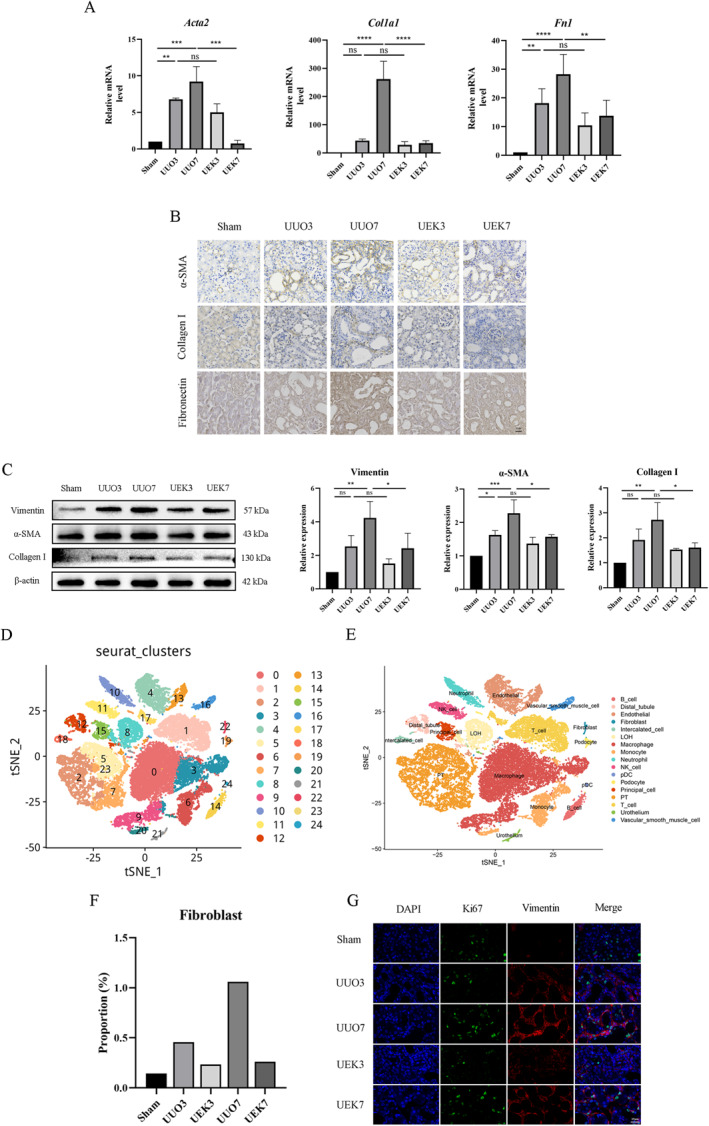
MSC‐Exo derived from human umbilical cord (HucMSC‐Exo) inhibits unilateral ureteral obstruction (UUO)‐induced renal fibrosis and fibroblast proliferation. (A) Real time quantitative PCR detection of mRNA expression levels of α‐SMA, collagen I, and fibronectin; (B) representative immunohistochemical images of fibrosis markers α‐SMA, collagen I, and fibronectin; (C) western blot analysis of protein expression levels of vimentin, α‐SMA, and collagen I in kidneys from each group, along with quantitative analysis; (D–E) samples from different treatments were integrated into a single dataset, tSNE graph was used to visualize different clusters and cell types; (F) line chart showing the proportion of fibroblasts in each group of cells; (G) representative images of immunofluorescence colocalization of Ki67 and vimentin. *p* > 0.05; *p < 0.05; **p < 0.01; ***p < 0.001; ****p < 0.0001. Distal_tubule, Distal convoluted tubule cell; LOH, Loop of Henle cell; MSC‐Exo, Exosomes derived from mesenchymal stem cells; NK_cell, Natural killer cell; ns, not significant‌; pDC, Plasmacytoid dendritic cell; PT, Proximal tubule cell; UEK3, UUO3+Exo; UEK7, UUO7+Exo.

Masson's trichrome staining results revealed minimal collagen deposition in UUO renal interstitium at day 3, progressing to extensive accumulation by day 7. Complementary analysis of fibrotic markers (α‐SMA, collagen I, and fibronectin) via real time‐qPCR (RT‐qPCR) demonstrated significant up‐regulation in UUO kidneys, all of which were markedly suppressed by HucMSC‐Exo treatment in the 7‐day model (Figure 2A). Consistent findings were observed by immunohistochemistry and western blot (Figure 2B,C). HucMSC‐Exo intervention substantially mitigated both the intensity and distribution of this fibrotic deposition (Figure 1D).

Single‐cell RNA sequencing of all five model groups identified 25 transcriptionally distinct clusters by unsupervised cluster analysis. Annotation using established marker genes defined 17 renal cell types, including macrophages, proximal tubules (PT) cells, podocytes, fibroblasts, and T cells (Figure 2D,E). Fibroblast‐specific analysis revealed a progressive expansion of this population in UUO kidneys, a trend reversed by HucMSC‐Exo administration (Figure 2F). This indicated HucMSC‐Exo‐mediated inhibition of injury‐induced fibroblast proliferation, corroborated by vimentin/Ki67 dual‐label immunofluorescence (Figure 2G). Collectively, these data demonstrate HucMSC‐Exo effectively modulates fibroblast dynamics and fibrotic progression in vivo.

### 3.3 HucMSC‐Exo Effectively Inhibits TGF‐β‐Induced NRK‐49F Cell Activation and Fibrosis

**FIGURE 3:**
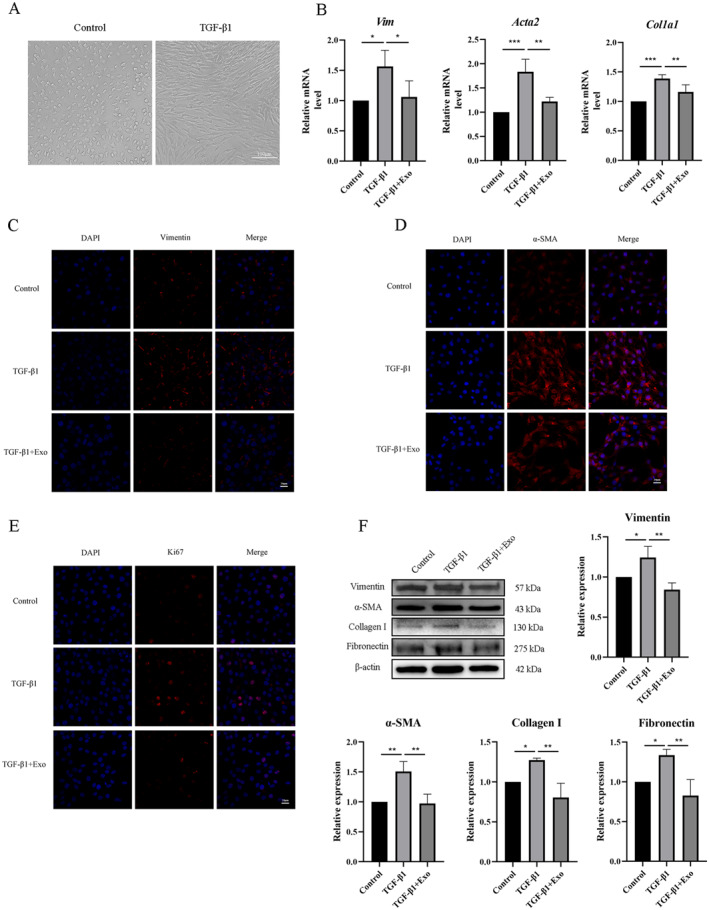
MSC‐Exo derived from human umbilical cord (HucMSC‐Exo) inhibits TGF‐β1‐induced proliferation of NRK‐49F cells and fibrosis in vitro. (A) Morphological changes of rat renal fibroblasts (NRK‐49F) in different groups observed under a microscope; (B) real‐time quantitative polymerase chain reaction (RT‐qPCR) analysis of mRNA changes in fibrosis markers vimentin, α‐SMA, and collagen I in vitro groups; (C–E) representative images of immunofluorescence detection of vimentin, α‐SMA, and Ki67; (F) western blot analysis of protein expression and quantitative analysis of fibrosis‐related markers. *p < 0.05; **p < 0.01; ***p < 0.001. MSC‐Exo, Exosomes derived from mesenchymal stem cells.

We further investigated whether HucMSC‐Exo could similarly suppress fibroblast proliferation and activation under in vitro conditions. To model fibrotic activation, NRK‐49F cells were stimulated with TGF‐β1, with or without HucMSC‐Exo treatment. Morphological assessment revealed that untreated NRK‐49F cells typically exhibited a polygonal or spike‐shaped morphology. However, following TGF‐β1 exposure, cells underwent significant elongation and thinning of the cytoplasm, adopting a more pronounced spindle‐shaped or elongated spike‐like appearance with disordered cellular arrangement (Figure 3A). At the molecular level, RT‐qPCR analysis demonstrated substantial upregulation of key myofibroblast differentiation markers and extracellular matrix components in TGF‐β1‐treated cells. This pathogenic induction was significantly attenuated by HucMSC‐Exo treatment (Figure 3B). Consistent suppression at the protein level was confirmed through immunofluorescence staining and western blot analysis (Figure 3C,D,F). Furthermore, immunofluorescence detection of the proliferation marker Ki67 revealed a parallel reduction in proliferative activity following HucMSC‐Exo intervention (Figure 3E), corroborating the morphological and biochemical findings. Collectively, these in vitro results demonstrate that HucMSC‐Exo effectively inhibits TGF‐β1‐induced proliferation, myofibroblast activation, and fibrotic transformation in NRK‐49F cells.

### 3.4 HucMSC‐Exo Significantly Reduces INHBA Expression in an Ex Vivo Fibrosis Model

**FIGURE 4:**
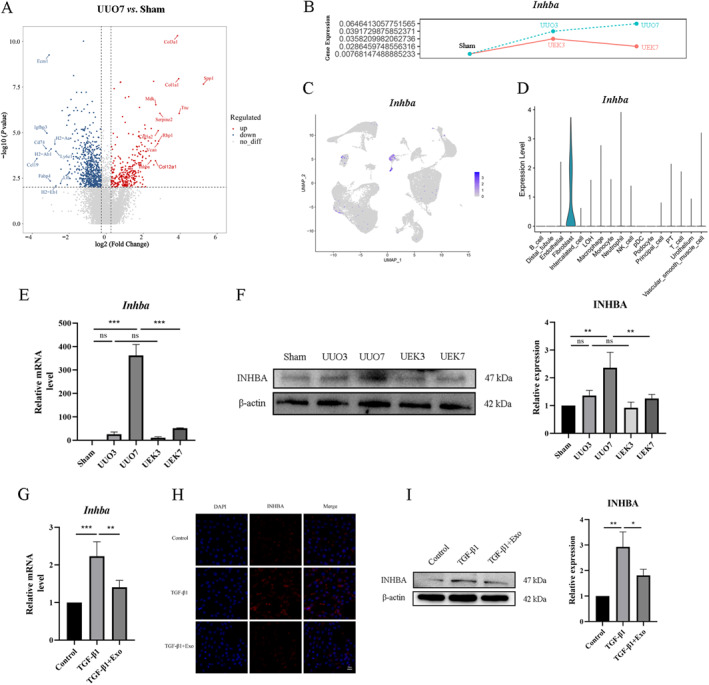
Expression of INHBA in in vivo and in vitro models. (A) Volcano plot of differentially expressed genes between the sham group and the UUO7 group; (B) line chart of *Inhba* gene expression in various in vivo models; (C, D) Uniform manifold approximation and projection (UMAP) plot and violin plot of *Inhba* gene expression in various cell types; (E) real‐time quantitative polymerase chain reaction (RT‐qPCR) analysis of mRNA changes in *Inhba* across different in vivo groups; (F) western blot analysis of INHBA protein expression in in‐vivo groups; (G) RT‐qPCR analysis of *Inhba* mRNA changes in various in vitro groups; (H, I) immunofluorescence and western blot detection of INHBA protein levels in vitro. *p* > 0.05; *p < 0.05; **p < 0.01; ***p < 0.001. Distal_tubule, Distal convoluted tubule cell; LOH, Loop of Henle cell; NK_cell, Natural killer cell; ns, not significant‌; pDC, Plasmacytoid dendritic cell; PT, Proximal tubule cell.

Previous studies have shown that activin A is involved in the processes of testicular and cardiac fibrosis [26, 27]. In the kidneys, activin A promotes the activation of fibroblasts, but the exact mechanism remains unclear. Comparative analysis of differential gene expression between the UUO 7‐day fibrosis model and Sham controls revealed significant upregulation of *Inhba* in fibrotic kidneys as visualized by the volcano plot (Figure 4A). Subsequent temporal evaluation demonstrated progressively elevated *Inhba* levels throughout UUO disease progression, an increase that was substantially attenuated by HucMSC‐Exo intervention (Figure 4B). Cellular localization analysis indicated *Inhba* expression was predominantly localized to fibroblasts within the renal compartment (Figure 4C,D). Molecular validation through RT‐qPCR confirmed pronounced elevation of *Inhba* transcript levels specifically in the 7‐day UUO model, with HucMSC‐Exo treatment significantly suppressing this induction. Western blot analysis corroborated these findings at the protein level, showing parallel reduction of INHBA expression following exosome administration (Figure 4E,F). The in vitro fibrotic model recapitulated these observations: TGF‐β1 stimulation significantly upregulated both mRNA and protein expression of INHBA in NRK‐49F fibroblasts. HucMSC‐Exo cotreatment effectively counteracted this pathogenic upregulation (Figure 4G–I). Collectively, these complementary in vivo and in vitro findings demonstrate that HucMSC‐Exo specifically targets and reduces INHBA expression within fibroblasts under profibrotic conditions.

### 3.5 HucMSC‐Exo Slows Down Fibrosis by Inhibiting INHBA Expression

**FIGURE 5:**
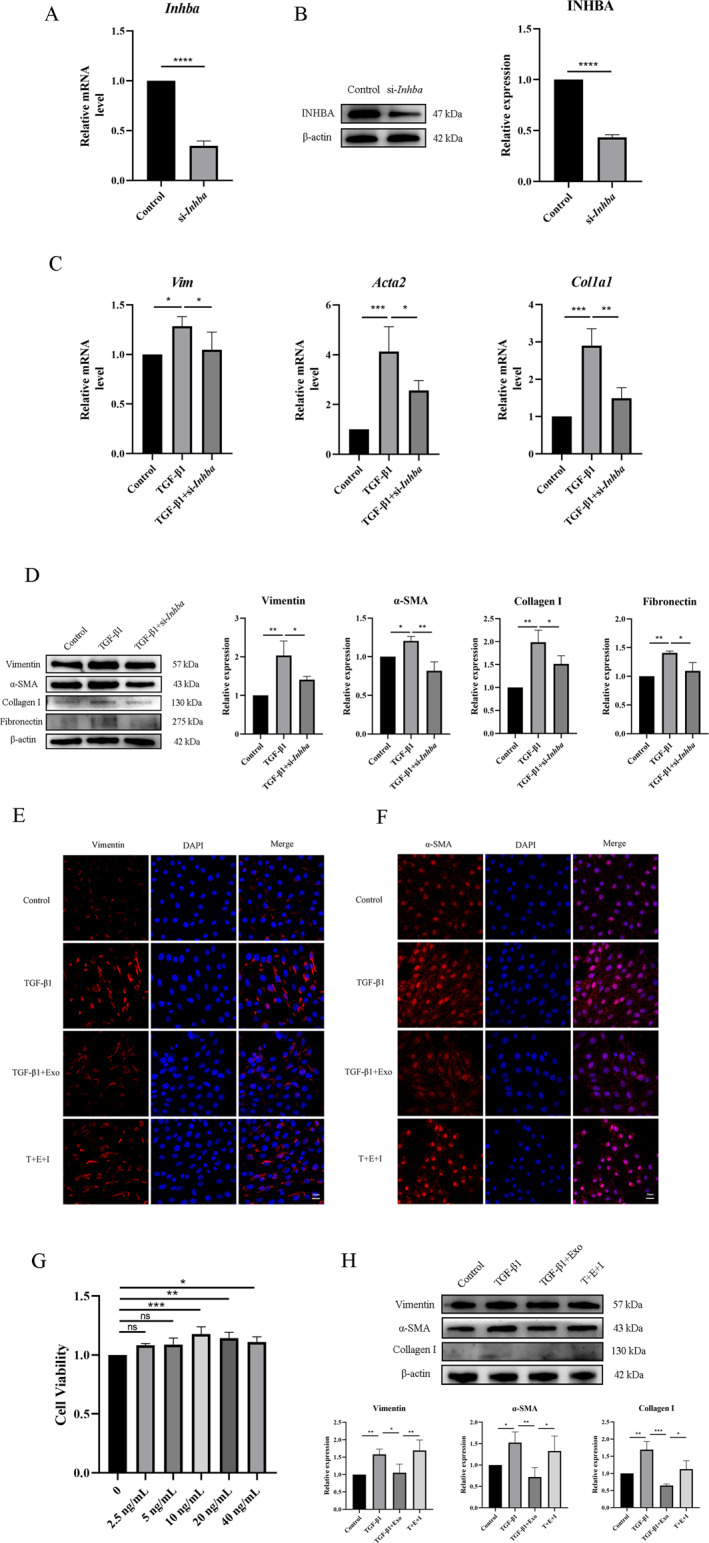
MSC‐Exo derived from human umbilical cord (HucMSC‐Exo) alleviates fibroblast activation and extracellular matrix deposition by inhibiting INHBA expression. (A) mRNA expression of the *Inhba* gene after siRNA knockdown in NRK‐49F cells; (B) protein expression of the *Inhba* gene after siRNA‐mediated knockdown in NRK‐49F cells; (C) mRNA expression of fibrosis‐related markers vimentin, α‐SMA, and collagen I after *Inhba* gene knockdown; (D) western blot analysis of protein expression of vimentin, α‐SMA, collagen I, and fibronectin; (E–F) immunofluorescence analysis of protein expression differences of vimentin and α‐SMA among different groups; (G) CCK‐8 assay of cell viability in NRK‐49F cells after adding different concentrations of exogenous INHBA; (H) western blot analysis of protein expression of fibrosis‐related markers among different groups. T + E + I: TGF‐β + Exo + INHBA. *p* > 0.05; *p < 0.05; **p < 0.01; ***p < 0.001; ****p < 0.0001. MSC‐Exo, Exosomes derived from mesenchymal stem cells; ns, not significant‌.

To elucidate the functional contribution of INHBA to fibrotic processes, we performed targeted gene knockdown in NRK‐49F cells using *Inhba*‐specific siRNA. Transfection efficiency analysis confirmed substantial reduction of *I* *nhba* expression at transcript level and INHBA expression at protein level, validating effective silencing (Figure 5A,B). Crucially, *Inhba* depletion significantly attenuated TGF‐β1‐induced upregulation of key fibrotic markers, including vimentin, α‐SMA, and collagen I, as evidenced by reduced mRNA expression and corresponding protein abundance (Figure 5C,D). These findings establish INHBA as a critical mediator of fibroblast activation during fibrogenesis.

To further investigate whether HucMSC‐Exo exerts its antifibrotic effects through INHBA regulation, we conducted rescue experiments with exogenous INHBA. Dose–response assessment via CCK‐8 assay identified 10 ng/mL as the optimal concentration for maintaining NRK‐49F viability (Figure 5G). This concentration was subsequently administered to TGF‐β + Exo group cells. Strikingly, supplemental INHBA partially reversed the anti‐fibrotic effects of HucMSC‐Exo, elevating protein expression of vimentin, α‐SMA, and collagen I as visualized by immunofluorescence and quantified by immunoblotting (Figure 5E,F,H). This reversal demonstrated that HucMSC‐Exo attenuated TGF‐β1‐driven fibrosis primarily through suppression of INHBA signaling.

### 3.6 Knockdown of *Inhba* Inhibits Activation of PI3K‐AKT Pathway in NRK‐49F Cells

**FIGURE 6:**
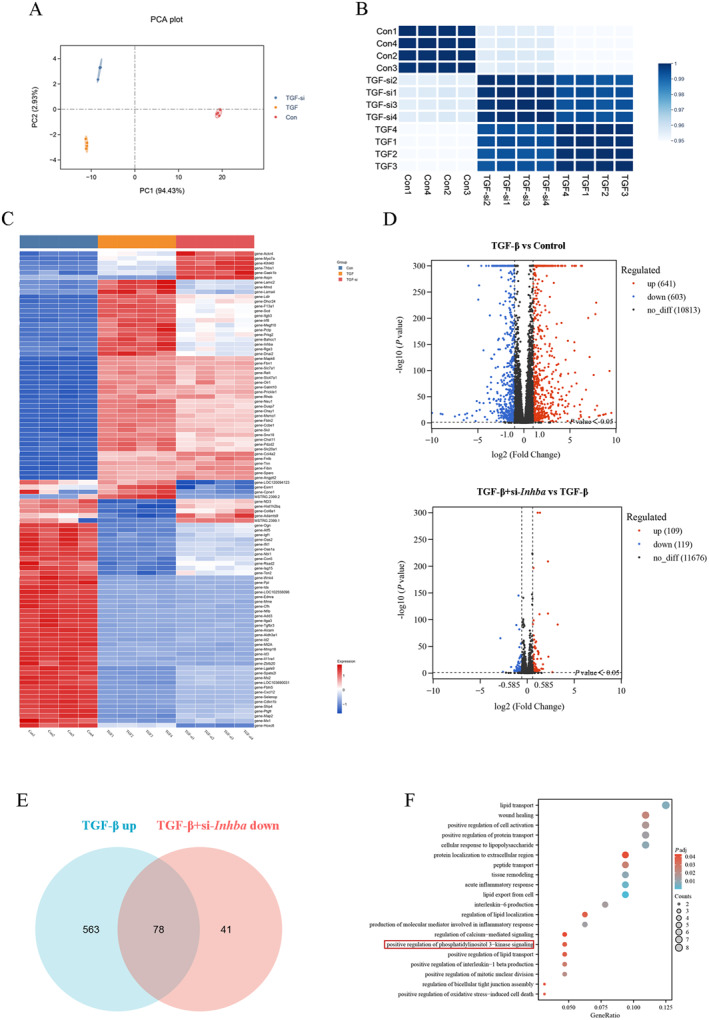
Transcriptome sequencing results analyzed using bioinformatics. (A) Principal component analysis (PCA) plot of the three groups: control, TGF‐β, and TGF‐β + si*‐Inhba*; (B) heat map showing gene expression levels in the three groups: control, TGF‐β, and TGF‐β + si*‐Inhba*; (C) gene expression heat map of the three groups: control, TGF‐β, and TGF‐β + si*‐Inhba*; (D) volcano plot of differentially expressed genes between control and TGF‐β (top) and between TGF‐β and TGF‐β + si*‐Inhba* (bottom); (E) Venn diagram showing overlap between TGF‑β‑induced upregulated genes and si‑*Inhba*‑reversed genes. Left circle (blue): Genes upregulated in TGF‑β versus Control; Right circle (red): Genes downregulated in TGF‑β + si‑*Inhba* versus TGF‑β; (F) Gene Ontology (GO) enrichment analysis of the intersecting genes in Figure (E).

On the basis of the above results, we carried out an in‐depth exploration of the mechanism that underlies INHBA action in the fibrotic process. We performed transcriptome sequencing on cells from the three groups of control, TGF‐β1, and TGF‐β1 + si*‐Inhba*. Principal component analysis and correlation analysis showed significant transcriptomic differences between the three groups of samples with high intragroup correlation (Figure 6A,B), and cluster analysis also showed significant differences in gene expression between the groups (Figure 6C). We used volcano plots to visualize the differentially expressed genes between groups, and a total of 1244 differentially expressed genes were identified between the control and TGF‐β1 groups, whereas 228 DEGs were identified in the TGF‐β1 and TGF‐β1 + si*‐Inhba* groups (Figure 6D). Notably, established profibrotic markers *Acta2*, *Col1a1*, *Pdgfa*, and *Postn* exhibited significant upregulation following TGF‐β induction (Supporting Information S1: Figure S1A). Functional annotation of control‐TGF‐β differentially expressed genes revealed coordinated pathway activation: GO analysis demonstrated enrichment in inflammatory response and extracellular matrix organization; KEGG mapping identified ECM‐receptor interaction, cytokine‐cytokine receptor signaling, PI3K‐Akt activation, Hippo transduction, and TNF signaling; whereas GSEA confirmed activation of inflammation, extracellular matrix remodeling, PI3K‐AKT cascade, NF‐κB signaling, WNT pathway, and NOTCH‐mediated transcription in TGF‐β‐treated cells (Supporting Information S1: Figure S1B–D). This transcriptional profiling conclusively demonstrates TGF‐β orchestrates fibrotic reprogramming through integrated signaling network activation.

Comparative transcriptomic analysis revealed 641 significantly upregulated genes in TGF‐β‐stimulated NRK‐49F cells versus controls. Following *Inhba* knockdown in the TGF‐β background, 119 genes exhibited substantial downregulation relative to TGF‐β‐treated cells (Figure 6D). Intersectional analysis identified 78 overlapping transcripts directly regulated by *Inhba* (Figure 6E). Functional annotation of these coregulated genes through GO analysis revealed significant enrichment in the PI3K signaling cascade (Figure 6F, Supporting Information S1: Figure S1E), implicating INHBA as a key modulator of PI3K pathway activation during profibrotic transformation.

### 3.7 HucMSC‐Exo Attenuates Fibrosis by Inhibiting the PI3K‐AKT Pathway via INHBA

**FIGURE 7:**
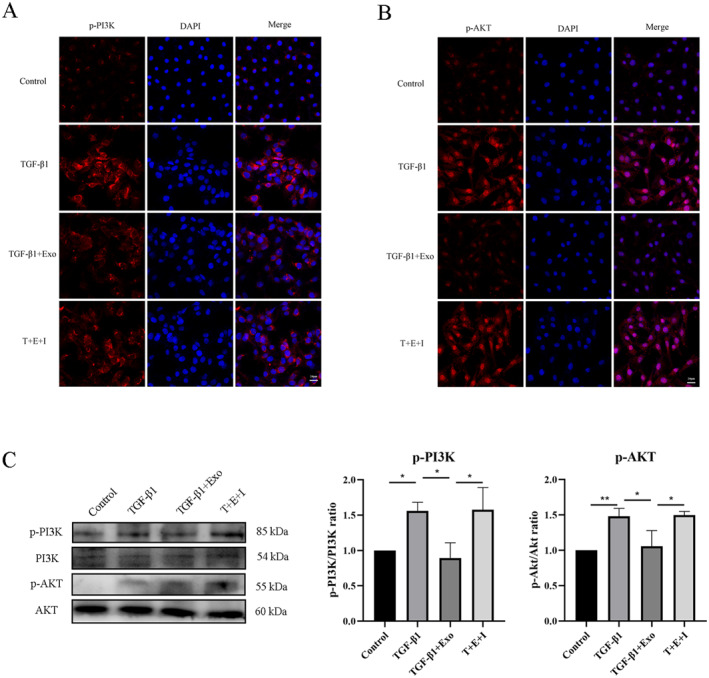
MSC‐Exo derived from human umbilical cord (HucMSC‐Exo) inhibits the activation of the PI3K/AKT signaling pathway in vitro. (A, B) Representative images of immunofluorescence detection of phosphorylated PI3K and AKT, respectively; (C) western blot detection of protein expression of phosphorylated PI3K and AKT in each group. T + E + I: TGF‐β + Exo + INHBA. *p < 0.05; **p < 0.01. MSC‐Exo, Exosomes derived from mesenchymal stem cells.

Extensive prior research has established PI3K/AKT signaling as a conserved mediator of fibrotic pathogenesis across multiple organ systems. Building upon our transcriptomic data revealing PI3K/AKT pathway activation in TGF‐β‐stimulated cells, we hypothesized that HucMSC‐Exo attenuates fibrosis through sequential inhibition of INHBA expression and consequent suppression of PI3K/AKT signaling. To validate this mechanistic model, we quantified phosphorylation states of core PI3K/AKT pathway components across experimental groups. Immunofluorescence analysis demonstrated significantly elevated p‐PI3K and p‐AKT levels in TGF‐β‐treated cells, consistent with pathway hyperactivation. HucMSC‐Exo intervention substantially reduced this phosphorylation, whereas concurrent INHBA supplementation attenuated exosome‐mediated suppression (Figure 7A,B). Western blotting corroborated these findings, showing identical phosphorylation patterns at the protein level (Figure 7C). This orthogonal validation confirms HucMSC‐Exo disrupts the INHBA/PI3K/AKT signaling axis to exert antifibrotic effects.

## 4 Discussion

The severity, duration, and frequency of AKI are critical determinants of maladaptive renal repair, which can manifest as tubular atrophy and interstitial fibrosis, ultimately driving the transition from AKI to CKD and, in severe cases, ESRD. This trajectory imposes an enormous socioeconomic burden worldwide. Among these pathological features, interstitial fibrosis remains the central hallmark of progressive kidney disease. Fibrosis represents not only an excessive accumulation of ECM but also a complex and dynamic process involving aberrant activation of signaling pathways, persistent inflammation, cytokine release, and intricate cell–cell interactions [28, 29]. The lack of clinically approved antifibrotic drugs underscores the urgency of identifying novel molecular mechanisms and therapeutic targets.

In this study, we utilized single‐cell RNA sequencing technology to identify the key regulatory gene involved in fibrosis—*Inhba*. Further studies confirmed that HucMSC‐Exo effectively inhibited fibroblast activation in UUO models and TGF‐β‐induced conditions by suppressing INHBA expression. Combined with transcriptomic sequencing analysis, we elucidated the specific molecular mechanisms by which INHBA drives fibroblast activation and promotes fibrosis through the PI3K/AKT signaling pathway. Collectively, these findings highlight a previously unrecognized role of HucMSC‐Exo in regulating fibroblast biology and provide mechanistic insights into their antifibrotic potential.

Stem cell‐based therapies, particularly MSCs, have been widely investigated for organ repair and regeneration due to their multipotent differentiation capacity. They have demonstrated therapeutic potential in multiple disease models, including various cancers (such as breast cancer and bladder cancer), acute and chronic lung diseases, and liver diseases [30, 31, 32]. Our previous studies have also confirmed that MSCs can effectively reduce the degree of fibrosis in various renal fibrosis models [33, 34]. Despite encouraging preclinical data, clinical translation of MSC therapy faces challenges, including safety concerns, tumorigenicity, and heterogeneity, in therapeutic efficacy. Increasing evidence suggests that the paracrine actions of MSCs, mediated largely through extracellular vesicles, such as exosomes, are the primary drivers of their beneficial effects. MSC‐Exo retains the bioactivity of parental cells while offering advantages such as higher safety, lower immunogenicity, lower risk of rejection, and better scalability. Importantly, MSC‐Exo has been shown to modulate diverse processes including proliferation, immune regulation, and autophagy, and even to serve as delivery vehicles for therapeutic RNAs and drugs [35, 36].

In the present work, HucMSC‐Exo treatment significantly ameliorated renal morphological injury, inflammation, fibroblast proliferation, and ECM deposition in UUO‐induced renal fibrosis. The therapeutic effects were time‐dependent, showing limited efficacy during the early phase (third day after obstruction), consistent with the absence of substantial fibrotic remodeling at this stage. Although the UEK7 group received two injections, its IL‐1β level remained higher than that of the UEK3 group, suggesting that prolonged ureteral obstruction can lead to pathological changes that are difficult to fully reverse, further emphasizing the importance of early intervention. In both in vitro and in vivo models, fibroblast proliferation and myofibroblast activation as marked by Ki67 and α‐SMA expression, respectively, were markedly suppressed by HucMSC‐Exo. These results strongly support their role in disrupting the fibroblast‐to‐myofibroblast transition and ECM overproduction—core drivers of fibrosis.

TGF‐β is one of the key factors driving the onset and progression of fibrosis. Activin A (INHBA) is a member of the TGF‐β superfamily too, composed of the inhibin βA subunit encoded by the *Inhba* gene, and is expressed in organs such as the testes, ovaries, and liver. In the kidneys, Activin A is primarily expressed during embryonic development, with extremely low expression in normal adult kidneys. However, its expression is notably upregulated following injuries such as ischemia/reperfusion. Research indicates that Activin A possesses multifunctional biological activities, including promoting tumor progression, cell proliferation, and fibrotic responses in various organs. For example, Activin A secreted by neurogenic fibroblasts promotes Schwann cell proliferation; and in the liver and heart, Activin A stimulates the proliferation and differentiation of hepatic stellate cells and cardiac fibroblasts through different mechanisms, respectively, exacerbating tissue remodeling and fibrosis [27, 37]. In this study, we found that INHBA is primarily expressed in renal fibroblasts and significantly upregulated during UUO‐induced fibrosis, whereas HucMSC‐Exo effectively attenuated its expression. Functional experiments validated the causal relationship: siRNA‐mediated *Inhba* knockdown alleviated TGF‐β‐induced fibrosis, whereas recombinant INHBA protein reversed the protective effects of HucMSC‐Exo. These results establish INHBA as a key downstream mediator in renal fibrosis and a direct therapeutic target of HucMSC‐Exo.

Previous studies indicated that Activin A promotes fibrosis in other organs through Smad3 or calcium signaling [38]. However, its role in renal fibroblasts remained poorly defined. Our transcriptomic and functional analyses demonstrated that INHBA activates the PI3K/AKT signaling pathway, a well‐established regulator of fibrotic responses across multiple organs [39, 40, 41]. Importantly, HucMSC‐Exo inhibited PI3K/AKT pathway activation, correlating with reductions in fibrosis markers, whereas recombinant INHBA restored pathway activation. These findings delineate a novel mechanistic axis in renal fibrosis: HucMSC‐Exo → suppression of INHBA → inhibition of PI3K/AKT signaling → attenuation of fibroblast activation and ECM deposition.

Despite these advances, this study still has certain limitations. First, exosomes are complex in composition and contain a wealth of bioactive molecules, including over 300 proteins and 150 microRNAs [42, 43]. This study has not yet identified which specific components mediate the primary antifibrotic effects. Second, this study focused on a UUO (obstructive) induced renal fibrosis model and lacks validation for fibrosis caused by other etiologies such as drug‐induced or ischemic fibrosis. Therefore, we will continue our research to explore the role and mechanisms of HucMSC‐Exo in renal fibrosis more comprehensively. Additionally, although this study has demonstrated the therapeutic potential of HucMSC‐Exo in renal injury and fibrosis, clinical application strategies (such as dosing regimens, scalable preparation, and safety assessment) remain key directions for future research.

## 5 Conclusions

This study demonstrates that HucMSC‐Exo exert significant antifibrotic effects by suppressing fibroblast activation and ECM deposition. Mechanistically, HucMSC‐Exo inhibit INHBA expression, thereby attenuating PI3K/AKT pathway activation and ameliorating renal fibrosis. These findings not only identify INHBA as a novel therapeutic target but also support the development of exosome‐based interventions as promising strategies for preventing and treating renal fibrosis in both acute and chronic kidney disease.

## Author Contributions

M.C. and C.J. participated in the conception and design of this study. M.C., C.J., and Z.S. participated in data collection and organization. B.Z. and Y.Y. performed statistical analysis. F.L. and J.C. provided technical guidance. M.C., X.L., T.X., Y.Z., D.Z., and G.W. participated in manuscript writing and rigorous revision. All authors have read and approved the final version of this manuscript.

## Funding

The research was supported by the Chongqing Key Project of Technology Innovation and Application (2024TIAD‐KPX0035), Joint Project of Chongqing Health Commission and Science and Technology Bureau (2025ZDXM038), and Chongqing Medical University Program for Youth Innovation in Future Medicine (W0056).

## Conflicts of Interest

The authors declare no conflicts of interest.

## Ethics Statement

The study was approved by the Animal Research Committee of Chongqing Medical University (no. CHCMU‐IACUC20250429004).

## Supporting information


Supporting Information S1

## Data Availability

The data that support the findings of this study are available from the corresponding author upon reasonable request.
