# Supplementary material for: Human Umbilical Cord Mesenchymal Stem Cell–Derived Exosomes Attenuate Renal Fibrosis by Suppressing Fibroblast Activation via the INHBA/PI3K/AKT Pathway
Source: Pediatr Discov. 2026 Jun 29:e70061. Online ahead of print. doi: 10.1002/pdi3.70061 (PMC13398571; doi:10.1002/pdi3.70061)
Supplement: Supplementary file 1 — Supporting Information S1 [file PDI3-9999-0-s001.docx]

**
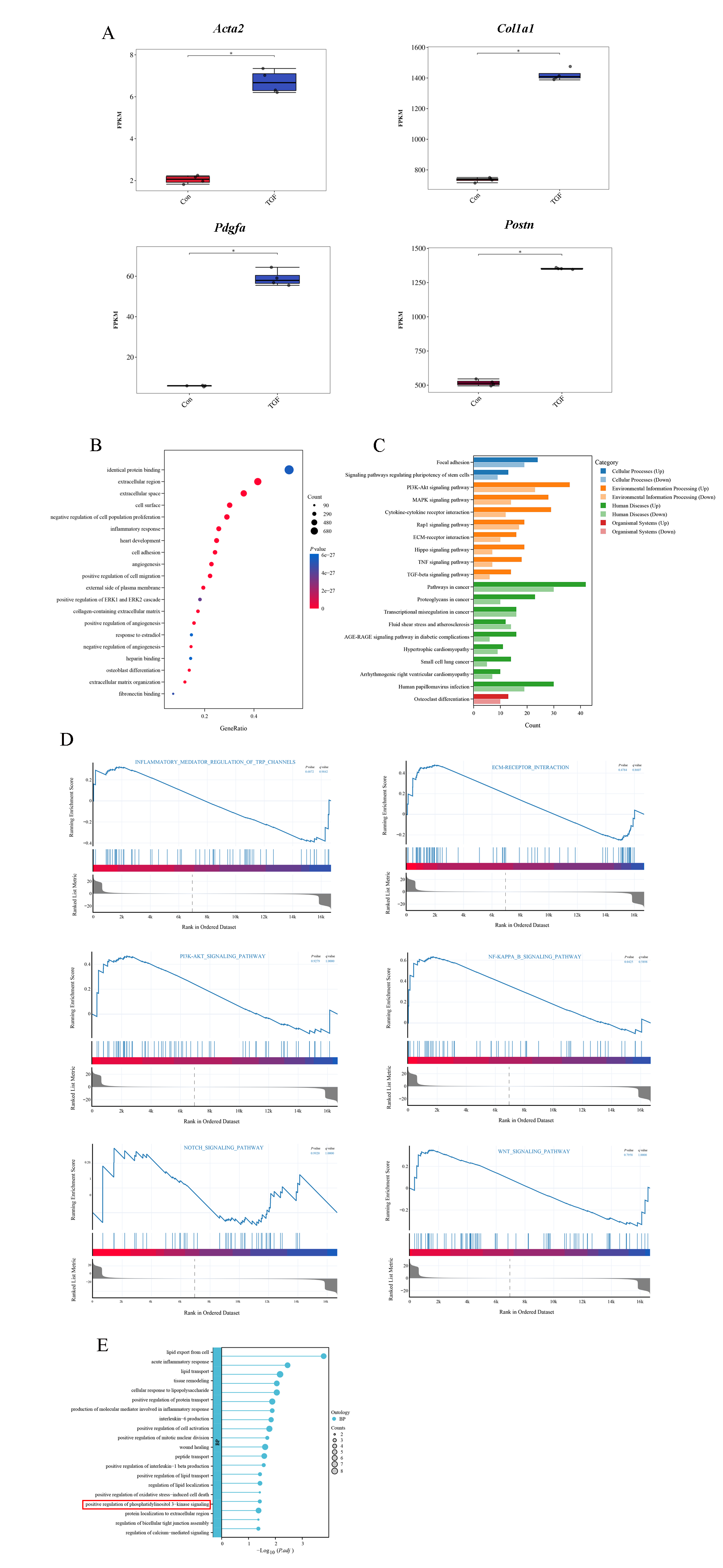
**

**Fig.S1.** Transcriptome sequencing-related results. (A) Box plots of the expression levels of the *Acta2*, *Col1a1*, *Pdgfa*, and *Postn* genes in the Control and TGF-β groups; (B-C) Gene Ontology (GO) and Kyoto Encyclopedia of Genes and Genomes (KEGG) enrichment analysis of differentially expressed genes between the Control and TGF-β groups; (D) Potential pathways of differentially expressed genes between the Control and TGF-β groups explored using Gene Set Enrichment Analysis (GSEA); (E) Lollipop plot of GO enrichment analysis for the intersecting genes in Figure 6E.

**Table.S1. The siRNA information of INHBA**

| **siRNA** | **Sense chain** | **Antisense chain** |
| --- | --- | --- |
| si-*Inhba*-1 | GUAGAUGCUCGGAAGAGUA | UACUCUUCCGAGCAUCUAC |
| si-*Inhba*-2 | GGAGAUAGAGGACGACAUU | AAUGUCGUCCUCUAUCUCC |
| si-*Inhba*-3 | CAACAGUCAUUAACCACUA | UAGUGGUUAAUGACUGUUG |

**Table.S2. RT-qPCR primer sequences**

| **Genes** | **Species** | **Forward primer sequence (5′–3′)** | **Reverse primer sequence (5′–3′)** |
| --- | --- | --- | --- |
| *Tgfb1* | Mouse | ACCGCAACAACGCCATCTATGAG | GGCACTGCTTCCCGAATGTCTG |
| *Il1b* | Mouse | CACTACAGGCTCCGAGATGAACAAC | TGTCGTTGCTTGGTTCTCCTTGTAC |
| *Acta2* | Mouse | CCCAGACATCAGGGAGTAATGG | TCTATCGGATACTTCAGCGTCA |
| *Col1a1* | Mouse | CTGGCGGTTCAGGTCCAAT | TTCCAGGCAATCCACGAGC |
| *Fn1* | Mouse | ATGTGGACCCCTCCTGATAGT | GCCCAGTGATTTCAGCAAAGG |
| *Inhba* | Mouse | AGAAGGGACCCGAAAGAGAATTT | GTCGTGTGGTTGCCTTCTTTAAA |
| *Gapdh* | Mouse | AGGTCGGTGTGAACGGATTTG | TGTAGACCATGTAGTTGAGGTCA |
| *Inhba* | Rat | AGAAGGGACCCGAAAGAGAATTT | GTCGTGTGGTTGCCTTCTTTAAA |
| *Vim* | Rat | GGATGTTGACAATGCTTCTCTGG | ATCTCTTCATCGTGCAGCTTCTT |
| *Acta2* | Rat | CCAGCTATGTGTGAAGAGGAAGA | TTGGTGATGATGCCGTGTTCTAT |
| *Col1a1* | Rat | GCCGCAAAGAGTCTACATGTCTA | AGGTTTCCACGTCTCACCATTAG |
| *Gapdh* | Rat | GAAGGTCGGTGTGAACGGAT | CCCATTTGATGTTAGCGGGAT |

**Table.S3.** **Primary antibodies**

| **Name** | **Catalog Number** | **Company** |
| --- | --- | --- |
| Anti-Vimentin | R22775 | Zenbio |
| Anti-α-SMA | 380653 | Zenbio |
| Anti-Collagen I | Bs-10423R | Bioss |
| Anti-Fibronectin | 15613-1-AP | Proteintech |
| Anti-Ki67 | R10018 | Zenbio |
| Anti-INHBA | HA500271 | HUABIO |
| Anti-p-PI3K | 341468 | Zenbio |
| Anti-PI3K | 67071-1-Ig | Proteintech |
| Anti-p-AKT | 66444-1-Ig | Proteintech |
| Anti-AKT | 10176-2-AP | Proteintech |
| Anti-β-actin | AC026 | ABclonal |
